# Supplementary figures and images for: Toward Early Diagnosis and Therapeutic Discovery in CLN3 Disease: A Computational Biomarker Discovery Framework
Source: medRxiv. 2026 May 7:2026.05.01.26352147. Preprint. [Version 1] doi: 10.64898/2026.05.01.26352147 (PMC13174741; doi:10.64898/2026.05.01.26352147)

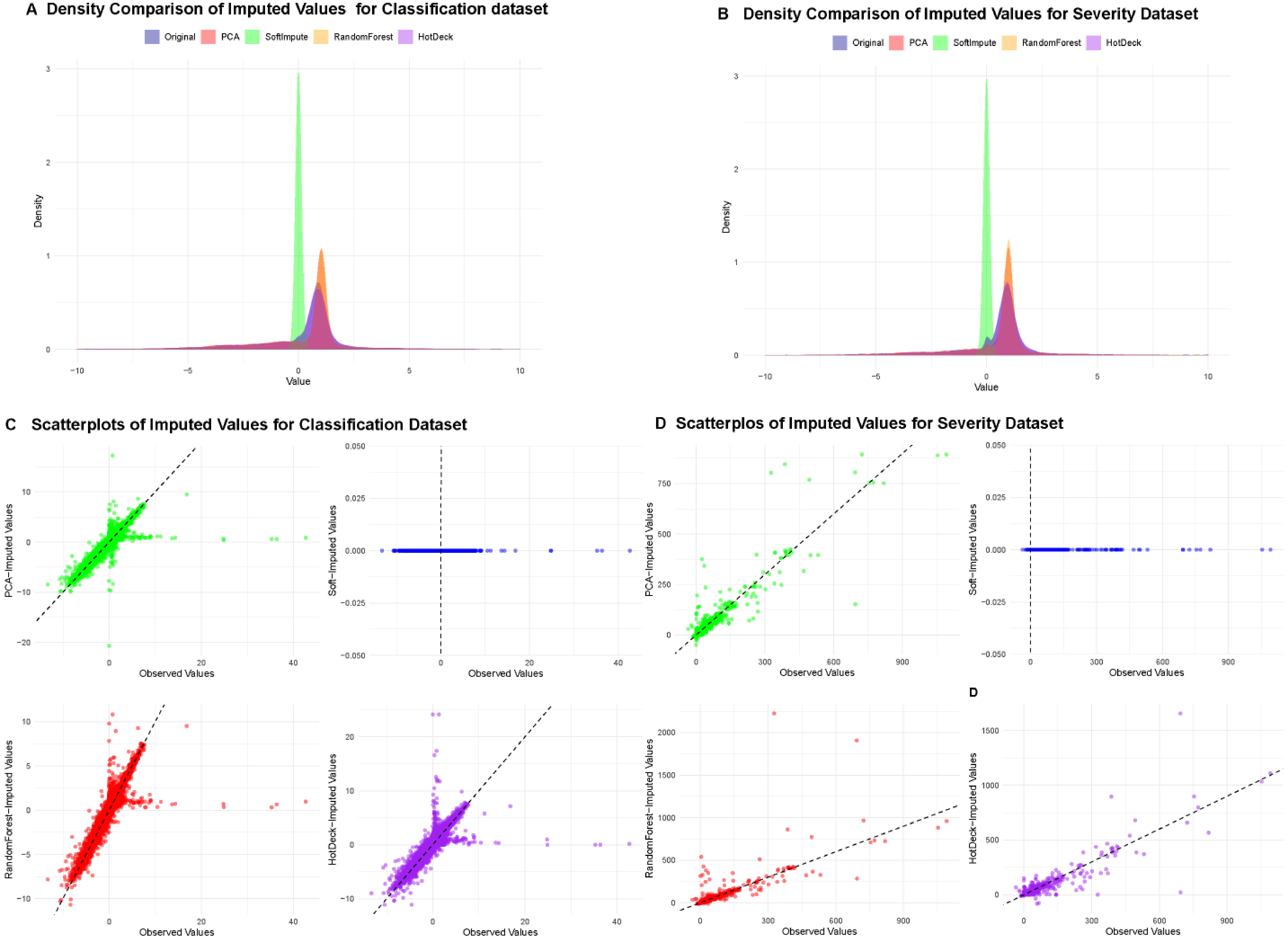

Supplement: Supplement 1 [file media-1.jpg]
