# Supplementary material for: Toward Early Diagnosis and Therapeutic Discovery in CLN3 Disease: A Computational Biomarker Discovery Framework
Source: medRxiv. 2026 May 7:2026.05.01.26352147. Preprint. [Version 1] doi: 10.64898/2026.05.01.26352147 (PMC13174741; doi:10.64898/2026.05.01.26352147)
Supplement: Supplement 2 [file media-2.pdf]

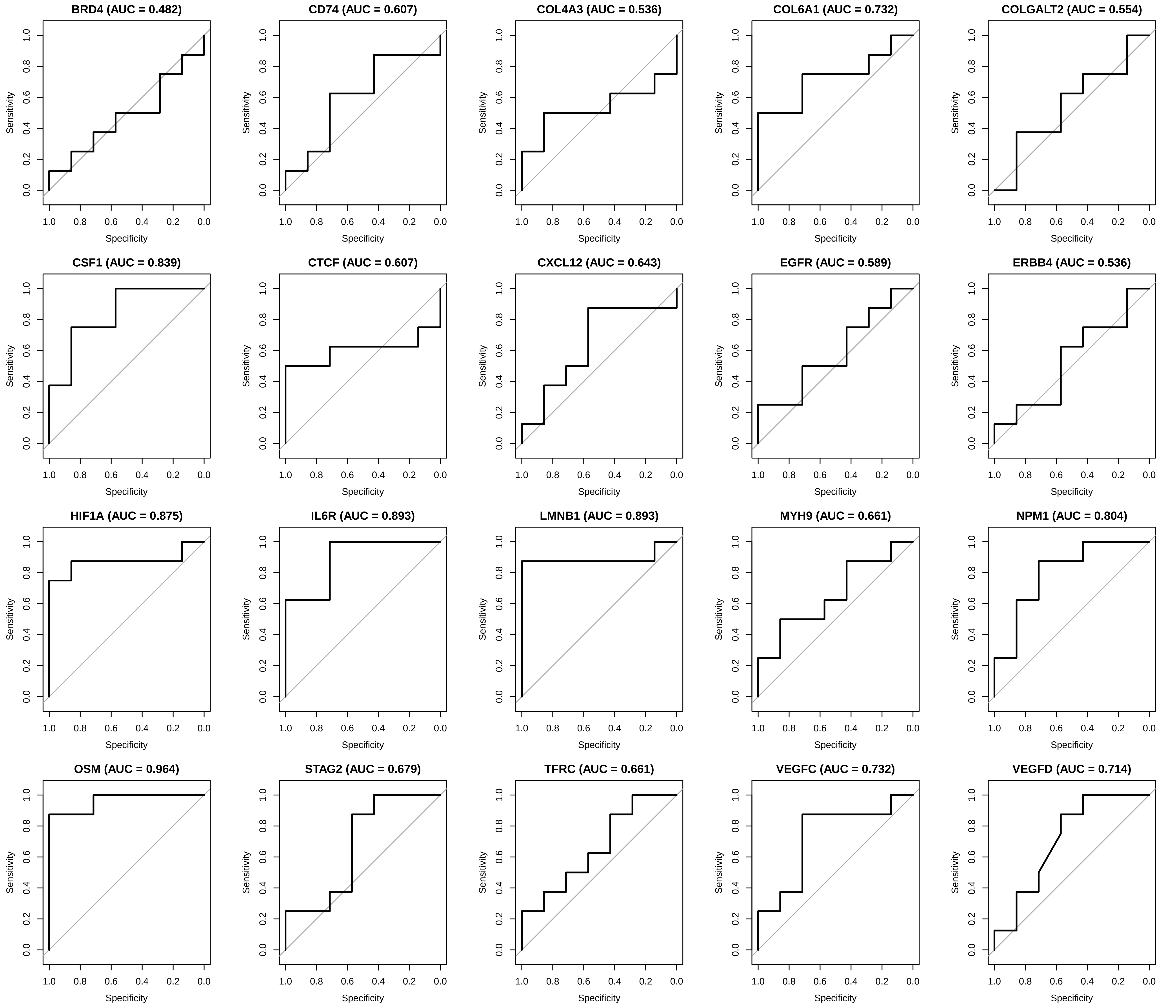
BRD4 (AUC = 0.482)
CD74 (AUC = 0.607)
COL4A3 (AUC = 0.536)
COL6A1 (AUC = 0.732)
COLGALT2 (AUC = 0.554)
CSF1 (AUC = 0.839)
CTCF (AUC = 0.607)
CXCL12 (AUC = 0.643)
EGFR (AUC = 0.589)
ERBB4 (AUC = 0.536)
HIF1A (AUC = 0.875)
IL6R (AUC = 0.893)
LMNB1 (AUC = 0.893)
MYH9 (AUC = 0.661)
NPM1 (AUC = 0.804)
OSM (AUC = 0.964)
STAG2 (AUC = 0.679)
TFRC (AUC = 0.661)
VEGFC (AUC = 0.732)
VEGFD (AUC = 0.714)
Sensitivity
Specificity
0.0
0.2
0.4
0.6
0.8
1.0
